# Proteomics and bioinformatics analyses based on two-dimensional electrophoresis and LC-MS/MS for the primary characterization of protein changes in chicken breast meat from divergent farming systems: Organic *versus* antibiotic-free

**DOI:** 10.1016/j.fochms.2024.100194

**Published:** 2024-01-17

**Authors:** Laura Alessandroni, Gianni Sagratini, Mohammed Gagaoua

**Affiliations:** a School of Pharmacy, Chemistry Interdisciplinary Project (CHIP), University of Camerino, Via Madonna delle Carceri, 62032 Camerino, Italy; b PEGASE, INRAE, Institut Agro, 35590 Saint-Gilles, France

**Keywords:** Chicken meat, Proteomics, Meat authenticity, Meat proteome, Biomarkers

## Abstract

Proteomics is a key analytical method in meat research thanks to its potential in investigating the proteins at interplay in post-mortem muscles. This study aimed to characterize for the first time the differences in early post-mortem muscle proteomes of chickens raised under two farming systems: organic *versus* antibiotic-free. Forty post-mortem *Pectoralis major* muscle samples from two chicken strains (Ross 308 *versus* Ranger Classic) reared under organic *versus* antibiotic-free farming systems were characterized and compared using two-dimensional electrophoresis and LC-MS/MS mass spectrometry. Within antibiotic-free and organic farming systems, 14 and 16 proteins were differentially abundant between Ross 308 and Ranger Classic, respectively. Within Ross 308 and Ranger Classic chicken strains, 12 and 18 proteins were differentially abundant between organic and antibiotic-free, respectively. Bioinformatics was applied to investigate the molecular pathways at interplay, which highlighted the key role of muscle structure and energy metabolism. Antibiotic-free and organic farming systems were found to significantly impact the muscle proteome of chicken breast meat. This paper further proposes a primary list of putative protein biomarkers that can be used for chicken meat or farming system authenticity.

## Highlights

• Two-dimensional electrophoresis to characterize the organic and antibiotic-free chicken breast muscle proteome.
• Discovery of protein biomarkers discriminating among chicken strains and farming systems.
• Impact of organic farming on chicken meat proteome among two strains.
• Bioinformatics to decipher the underlying biological mechanisms.

## 1 Introduction

Recent data suggest that the popularity of poultry meat continues to increase among consumers due to its affordability, availability, nutritional qualities and lower environmental impacts (Milford et al., 2019, González et al., 2020, Feknous et al., 2023). In fact, chicken is the mainly consumed poultry meat in Europe and its production has experienced a cumulative rise of around 30 % over the past 10 years (Augère-Granier, 2019). Commercial chicken strains have been selected predominately for individual performance traits such as breast percentage in carcasses and feed efficiency, with little regard to behavioral traits and adaptability of the animals to the farming system (Dal Bosco et al., 2021, Alessandroni et al., 2023).

Public demand regarding animal welfare and more humanely raised food, especially of animal origin, has grown exponentially over the recent years, pushing producers to shift from conventional and intensive production systems towards alternative and sustainable ones that are more focused on animal health and well-being (Alessandroni et al., 2022, Cooreman-Algoed et al., 2022). Among the extensive production systems, the inside ground farming with an antibiotic-free approach is a widespread system (Prache et al., 2021). In addition, organic animal production has experienced a rapid spread and development since the 1990s, which is driven, among other factors, by the willingness of consumers to pay for this kind of products. Indeed, outdoor access, for at least a third part of the lifespan, is one of the European guidelines for organic animal production requirements, together with organic feeding, the prohibition of antibiotics and synthetic compounds dispensation and other measures aiming maintaining high standards of animal welfare such as lighting, noise and ventilation (Commission, 834/2007, Commission, 889/2008). Studies have shown that chicken with outdoor access have improved plumage condition, and express less prevalence in bone deformities development and dermatitis (Bari et al., 2020, Wurtz et al., 2022).

The recent progress in food science and technology in the frame of foodomics brought by emerging analytical methods with novel and modern approaches allow better evaluations of food quality and safety. Foodomics is a powerful tool that can be used to investigate the molecular level of food nutrients and constituents (Balkir et al., 2021), including in muscle foods (Munekata et al., 2021). Proteomics has been one of the most appreciated and used foodomics approaches in the field of meat research (Gagaoua et al., 2024). Proteomics offers the analytical opportunity to apply effective quantitative methods for the characterization of the global or partial proteomes of the muscle tissues (Munekata et al., 2021, Gagaoua and Picard, 2022, Gagaoua and Zhu, 2022). For example, proteomics allows a better understanding of the biochemical mechanisms underpinning meat quality and authenticity (Gagaoua and Zhu, 2022, Gagaoua and Picard, 2022, Lamri et al., 2023b). The combination of electrophoresis separation, mainly two-dimensional electrophoresis, coupled with mass spectrometry (MS) methods is the most cost-efficient approach in proteomics as it allows the detection, characterization, and quantification of many proteins (Bonzon-Kulichenko et al., 2011, Meyer and Schilling, 2017, Picard and Gagaoua, 2020a, della Malva et al., 2023b, Scapol et al., 2023). The importance of proteomics in the field of meat research is illustrated by the large and ever increasing number of publications published during the last 20 years (Gagaoua and Picard, 2022, Gagaoua and Zhu, 2022). In poultry science, proteomics has been applied to investigate protein biomarkers related to chicken meat quality and safety traits (Phongpa-Ngan et al., 2011, Gagaoua et al., 2024). In fact, meat quality parameters such as texture, water holding capacity, cooking loss, pH, color, fat and mineral contents were investigated to understand their relationships with the proteomic profiles of chicken muscle (Phongpa-Ngan et al., 2011, Kuttappan et al., 2017, Cai et al., 2018, Zhang et al., 2020a, Zhang et al., 2020b). Moreover, several studies focused on chicken meat abnormalities like woody breast myopathy, white striping or pale, soft, and exudative (PSE) through different proteomic approaches (Desai et al., 2016, Soglia et al., 2016, Zambonelli et al., 2016, Zhang et al., 2020b). Although the popularity of organic and antibiotic-free management practices is on the rise, the impact of such methods on chicken muscle and meat proteome are still not studied.

This study aimed to apply for the first-time a proteomics approach, which consist on two-dimensional electrophoresis (2-DE) followed by liquid chromatography tandem mass spectrometry (LC-MS/MS) to assess differences in early post-mortem proteome between chicken breast meat from antibiotic-free and organic production systems and the impact of genotype investigated using two different chicken strains.

## 2 Materials and methods

### 2.1 Experimental design and muscle sampling

**Fig. 1:**
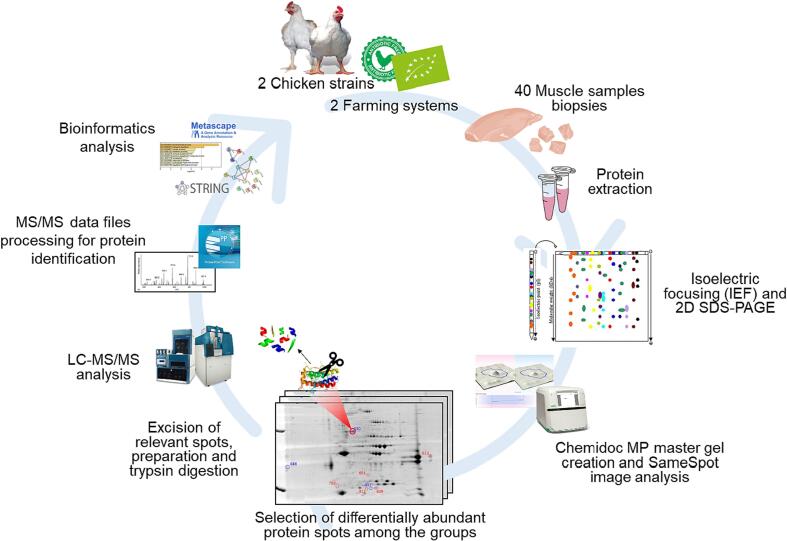
Workflow highlighting the steps followed in this study for the proteomics and bioinformatics analyses.

Forty chickens were used in this study and breast muscles were sampled at Fileni® industry (Cingoli, Italy). The samples consist of fresh meat sampled early post-mortem (3 h) following traditional industrial processes. The overall view of the four groups according to the chicken strain and farming system is reported in Table 1. Organic chickens were reared under the same conditions and farm according to the European Commission Regulation No 848/2018 for organic systems for poultry and livestock production, using organic feed, controlled housing, having access to an outdoor area with the presence of pasture for at least one third of their life. Antibiotic-free chickens were also of the same origin and featured a standard broiler inside-ground farming system, using concentrated feed and controlled housing (artificial light and climate control, automatic water, and feed supply) according to the European Directive 2007/43/EC. In this study, *Pectoralis major* muscles biopsies were taken immediately after slaughter under the same conditions by cutting a 2 cm^3^ section from the top right part of each breast with randomization. Samples were quickly frozen in liquid nitrogen and stored at −80 °C until proteomics analyses. The detailed workflow of the proteomics approach followed in this study is depicted in Fig. 1.

**Table 1:** Summary of the four experimental groups of analyzed samples and main animal characteristics.

Variables	Antibiotic-free system		Organic system	
Chicken strain	Ross 308	Ranger Classic	Ross 308	Ranger Classic
Number of samples	10	10	10	10
Sample name	ARO	ARA	ORO	ORA
Age	48 days	56 days	83 days	85 days
Sex	Male	Male	Female	Female
Average live weight	3.47 kg	3.22 kg	3.96 kg	3.00 kg

### 2.2 Protein extraction and quantification

For protein extraction, 200 mg of frozen muscle tissue samples were extracted using 3 mL of a fresh buffer containing 2 M thiourea, 8.3 M urea, 1 % Dithiothreitol, 2 % CHAPS (3-[(3-cholamidopropyl) dimethylammonio]-1-propanesulfonate) and 2 % Pharmalyte® (Immobilized pH gradient (IPG) buffer pH 3–10). The mixture kept at 4 °C under shaking for 15 min were then homogenized using a T 25 digital Ultra-Turrax® as previously described (Lamri et al., 2023a). Protein homogenates were incubated for 30 min in wet-ice and centrifuged for 30 min at 10000 rpm at 4 °C.

To have one reference sample for each group, 100 μL of each supernatant (x 10 samples) were pooled in an Eppendorf. The protein concentration of the pooled extracts were determined using the dye-binding protocol of Bradford (Bradford, 1976). A spectrophotometer (UV-1700, Pharmaspec, SHIMADZU) and bovine serum albumin as a standard (Bio-Rad Protein Assay kit, Bio-Rad, France) were used.

### 2.3 Two-dimensional electrophoresis (2-DE) and image analysis

For the separation of the proteins in two-dimensional SDS-PAGE gels, the methods previously described have been applied (Severino et al., 2022, della Malva et al., 2023a, della Malva et al., 2023b, Scapol et al., 2023). Briefly, 35 μg of each pooled sample was purified by MeOH/CHCl_3_ precipitation (to remove of interfering agents such as lipids, nucleic acids from the protein samples which aims to greatly enhance the resolution of proteins on acrylamide gels) and subsequently resuspended in 150 mL of DeStreak solution (Biorad Laboratories, Hercules) adding 1 % of IPG buffer solution (Biorad Laboratories, Hercules). The sample was loaded onto IPG strip (7-cm long, pH 3–10 linear gradient, ReadyStrip IPG strips, BioRad Laboratories, Hercules). Then, the first-dimension isoelectric focusing was performed using a PROTEAN IEF cell system (Bio-Rad Laboratories) for 20,000 total kVh at 20 °C adding 12 h of active hydration prior to the beginning of the voltage application. After equilibration, the second dimension was run on 10 % SDS-PAGE gel using a Mini-PROTEAN ® PrecastGels (Bio-Rad, USA). The gels were stained using SYPRO Ruby protein gel fluorescent stain (Lonza, Rockland) following the manufacturer’s indications.

The 2-DE images from gels (five gels replication for each treatment = 20 gels in total) stained with SYPRO Ruby fluorescent dyes were captured with the ChemiDoc MP Imaging System (Bio-Rad Laboratories) (della Malva et al., 2023a, della Malva et al., 2023b). In order to minimize the variability of the 2-DE method, a master gel was created using ChemiDoc MP Imaging System. The obtained master gel represents a gel image comprising all the protein spots believed to be representative of the sample, although not necessarily present in all samples of the group (Ong & Pandey, 2001). The master gel of each group was then compared to the others according to the experimental design. Analysis of digitalized gel images was performed with SameSpots 5.1.012 (TotalLab Laboratories). Protein volumes of detected and matched spots over biological replicates were automatically measured by SameSpots software and manually validated. Only protein spots reproducibly detected in at the least three of five biological replicates were selected for image analyses. Thus, the mean of a minimum of three values was calculated for each sample and spot. The protein intensity in each spot was expressed as volume, *i.e.,* the sum of pixel intensity within the spot area. Differential abundance of a protein present in the gels was considered significant when the fold change was at least 1.2 and the *P*-value was below 0.05.

### 2.4 Protein identification by LC-MS/MS

#### 2.4.1 Protein digestion

To identify the proteins in the spots of interest, we cut manually and under careful conditions the spots that showed significant differences after densitometry analysis. The protein spots were then subjected to a tryptic digestion (Baldassini et al., 2022, Gagaoua and Picard, 2022, Gagaoua and Zhu, 2022). Before that, the spots were reduced and alkylated as in the following protocol: reduction with 10 mM dithiothreitol (Sigma-Aldrich, St. Louis, MO) in 50 mM ammonium bicarbonate (Sigma-Aldrich, St. Louis, MO) and alkylation with 55 mM iodoacetamide (Sigma- Aldrich, St. Louis, MO) in 50 mM ammonium bicarbonate. Then, modified porcine trypsin (Promega, Madison, WI, USA) at a final concentration of 20 ng/μL in 20 mM ammonium bicarbonate was added and digestion was carried out by incubating the samples at 37 °C for 16 h. Peptides were purified using a C18 columns. The resulting peptide extracts were pooled, concentrated in a SpeedVac and stored at − 20 °C until analysis.

#### 2.4.2 Mass spectrometry (LC-MS/MS) analysis

The digested peptides of each spot were separated using Reverse Phase Chromatography. A gradient has been developed using a micro liquid chromatography system (Eksigent Technologies nanoLC 400, SCIEX) coupled to high-speed Triple TOF 6600 mass spectrometer (SCIEX, Foster City, CA) with a micro flow source. The analytical column used was a silica-based reversed phase column YMC-TRIART C18 (150 × 0.30 mm), 3 µm particle size and 120 Å pore size (YMC Technologies, Teknokroma). The trap column was a YMC-TRIART C18 (YMC Technologies, Teknokroma) with a 3 µm particle size and 120 Å pore size, switched on-line with the analytical column. The loading pump delivered a solution of 0.1 % formic acid in water at 10 µL/min. The micro-pump provided a flow-rate of 5 µL/min and was operated under gradient elution conditions, using 0.1 % formic acid in water as mobile phase A, and 0.1 % formic acid in acetonitrile as mobile phase B. Peptides were separated using a 90 min gradient ranging from 2 % to 90 % mobile phase B (Anfray et al., 2021, Peñas-Martínez et al., 2021, Chantada-Vázquez et al., 2022). Data acquisition was carried out using a Data dependent workflow. Source and interface conditions were as follows: ion spray voltage floating 5500 V, curtain gas 25, collision energy 10 and ion source gas 1 25. The instrument was operated with Analyst TF 1.7.1 software (SCIEX, USA) and the switching criteria were set to ions greater than mass to charge ratio (*m*/*z*) 350 and smaller than *m*/*z* 1800 with charge state of 2–5, mass tolerance 250 ppm and an abundance threshold of more than 200 counts. Former target ions were excluded for 15 s. Instrument was automatically calibrated every 4 h using as external calibrant tryptic peptides from pepcalMix.

#### 2.4.3 Statistical and bioinformatics analyses on the LC-MS/MS data

After LC-MS/MS analysis, the data files were processed using ProteinPilotTM 5.0.1 software from Sciex which uses the algorithm Paragon^TM^ for database search and ProgroupTM for data grouping. Data were searched using a chicken (*Gallus gallus*) specific Uniprot database and by setting trypsin as enzyme used in the digestion, iodoacetamide to perform the Cys carboxyamidomethylation and as a special feature a lysine biotinylation. False discovery rate was performed using a non-lineal fitting method displaying only those results that reported a 1 % global false discovery rate (FDR) or better as previously specified (Shilov et al., 2007, Tang et al., 2008).

The bioinformatics analyses were performed on the protein lists (using gene names) to identify the main molecular and biological functions using Gene Ontology (GO) analyses to highlight the major and related molecular signatures, and protein–protein interaction (PPI) to build protein networks and highlight the degree interconnectedness of the proteins. For GO analyses, the Metascape® open-source tool (https://metascape.org/) was used to functionally categorize and identify the significant and enriched GO, and to investigate the pathways and process enrichments using the total differentially abundant proteins (DAPs). The tool combines a hypergeometric test and Benjamini − Hochberg p-value correction algorithm to display the first statistically significant enriched ontology terms. For PPI network analyses, the open-source STRING v11.0 database (https://string-db.org) was used to relate the protein lists. The analysis considered interactions that had a medium confidence score of 0.40.

## 3 Results

The results of the 2-DE gels (images) analysis revealed a total of 51 protein spots (for the whole experiment) of interest with statistically significant differences based on the fold change thresholds we used. The LC-MS/MS analysis allowed the characterization of the content of the protein spots in terms of proteins, named in this paper as DAPs. In the following sections, the results have been divided according to the pairwise comparisons performed within, and between, the two chicken strains and the two farming systems.

### 3.1 Antibiotic-free chicken meat samples: DAPs between chicken strains

**Fig. 2:**
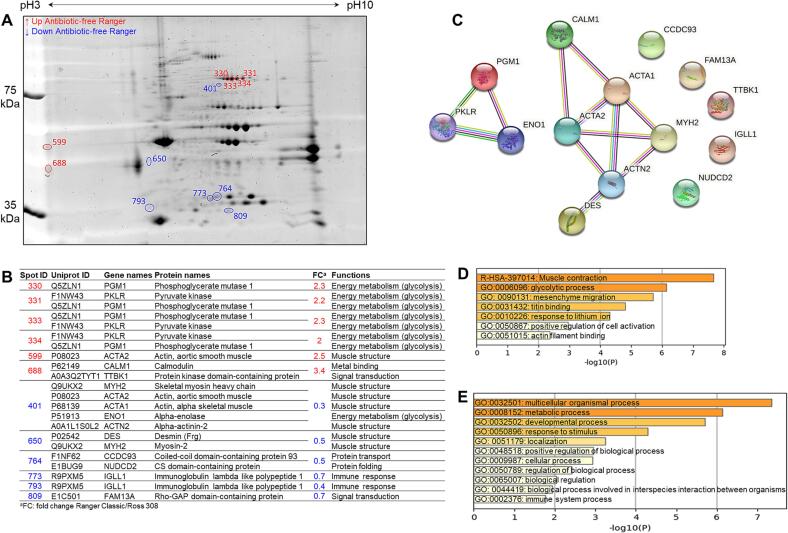
Comparison between the chicken breast muscle proteomes of Ross 308 and Ranger Classic within the antibiotic-free production system. A) Representative two-dimensional gel electrophoresis highlighting the differentially abundant protein spots between the two groups. Spots in blue color correspond to the down-regulated proteins, those in red are up-regulated proteins in Ranger Classic chicken meat. B) Identify of differentially abundant proteins (DAPs) including their functions and fold change. C) Protein-Protein interaction (PPI) network using the list of the 14 DAPs in antibiotic-free chicken breast muscle. The PPI network was built using the STRING database (https://string-db.org/). D-E) Bioinformatics enrichment analyses (Gene Ontology, KEGG, Reactome) using the 14 DAPs. D) Top enriched cluster terms. E) Biological processes. The enrichment analysis was performed using Metascape webservice tool (https://metascape.org/).

The 2-DE gel comparison of the samples of antibiotic-free Ross 308 (ARO) and Ranger Classic (ARA) is given in Fig. 2A. The densitometry analyses revealed 12 differentially abundant spots, from which the up-regulated proteins are shown in red and the down-regulated ones in blue, using Ranger strain as reference. The gene names, Uniprot ID, protein names and functions are reported in Fig. 2B together with their fold change values calculated from the 2-DE image analysis for each protein spot. Interestingly, several proteins have been identified with several proteoforms. From the results, it seemed that calmodulin (CALM1), actin (ACTA2), phosphoglycerate mutase 1 (PGM1) and pyruvate kinase (PKLR) were more abundant in ARA samples while immunoglobulin (IGLL1), rho-GAP domain-containing protein (FAM13A), desmin (DES) and myosin-2 (MYH2) were the main overabundant proteins in ARO samples. These proteins can be proposed as candidate biomarkers to discriminate Ranger Classic breast meat samples from those of Ross 308 chicken reared under antibiotic-free inside ground farming.

The PPI analysis highlighted two main networks (Fig. 2C). The larger one is related to muscle structure proteins, mainly formed by various actin members (ACTA1, ACTA2, ACTN2), MYH2, CALM1 and DES. The second network is related to energy metabolism and is composed by PGM1, alpha-enolase (ENO1) and PKLR. The GO enrichment analyses on the 14 unique proteins through Gene Ontology, KEGG and Reactome databases are given in Fig. 2D and Fig. 2E. It resulted that 7 GO cluster terms were significantly enriched mainly dominated by “muscle contraction (R-HSA-397014)” and “glycolytic process (GO:0006096)”. The proteins belong to 11 biological processes from which “multicellular organismal process (GO:0032501)”, “metabolic process (GO:0008152)” and “developmental process (GO:0032502)” as the top enriched ones.

### 3.2 Organic chicken meat samples: DAPs between chicken strains

**Fig. 3:**
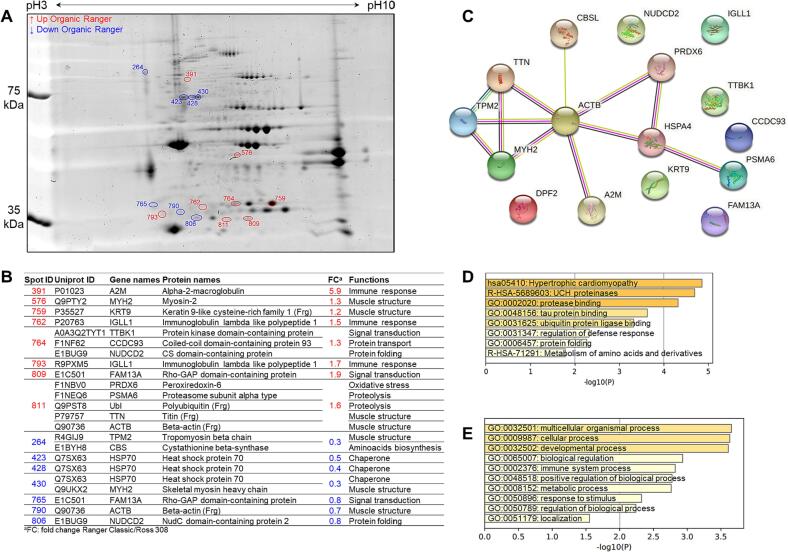
Comparison between the chicken breast muscle proteomes of Ross 308 and Ranger Classic within the organic production system. A) Representative two-dimensional gel electrophoresis highlighting the differentially abundant protein spots between the two groups. Spots in blue color correspond to the down-regulated proteins, those in red are up-regulated proteins in Ranger Classic chicken meat. B) Identify of differentially abundant proteins (DAPs) including their functions and fold change. C) Protein-Protein interaction (PPI) network using the list of the 16 DAPs in organic chicken breast muscle. The PPI network was built using the STRING database (https://string-db.org/). D-E) Bioinformatics enrichment analyses (Gene Ontology, KEGG, Reactome) using the 16 DAPs. D) Top enriched cluster terms. E) Biological processes. The enrichment analysis was performed using Metascape webservice tool (https://metascape.org/).

The 2-DE gel comparison of the samples of organic Ross 308 (ORO) and Ranger Classic (ORA) is given in Fig. 3A. The densitometry analyses revealed 15 differentially abundant spots, from which the up-regulated proteins are shown in red and the down-regulated ones in blue, using Ranger strain as reference. The gene names, Uniprot ID, protein names and functions are reported in Fig. 3B together with their fold change values calculated from the 2-DE image analysis for each protein spot. Interestingly, several proteins have been identified with several proteoforms. Among the most abundant proteins in ORA, alpha-2-macroglobulin (A2M) was the mainly DAP with a fold change score of 5.9 followed by IGLL1 and FAM13A. NudC domain-containing protein 2 (NUDCD2) and beta-actin (ACTB) were two of the most abundant proteins in ORO samples. These proteins can be also proposed as candidate biomarkers to discriminate Ranger Classic breast meat samples from those of Ross 308 chicken reared under organic farming system.

The PPI analysis highlighted one main network related to muscle structure pathways (Fig. 3C). Interestingly, the protein that has the highest number of interactions was ACTB with 7 interactions. The GO enrichment analyses on the 16 DAPs through Gene Ontology, KEGG and Reactome databases are given in Fig. 3D and Fig. 3E. It resulted that 8 GO cluster terms were significantly enriched mainly dominated by “hypertrophic cardiomyopathy (hsa:05410)”, “UCH proteinases (R-HSA-5689603)” and “protease binding (GO:0002020)”. Furthermore, 10 biological processes were enriched with “multicellular organismal process (GO:0032501)”, “cellular process (GO:0009987)” and “developmental process (GO:0032502)” as the top enriched biological processes terms.

### 3.3 Ross 308 chicken meat samples: DAPs between farming systems

**Fig. 4:**
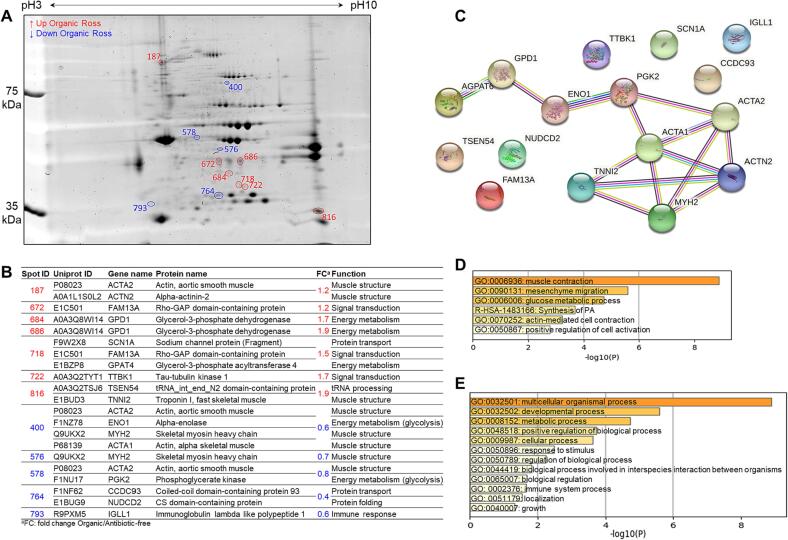
Comparison between antibiotic-free and organic chicken breast muscle proteomes within the Ross 308 strain. A) Representative two-dimensional gel electrophoresis highlighting the differentially abundant protein spots between the two groups. Spots in blue color correspond to the down-regulated proteins, those in red are up-regulated proteins in organic chicken meat. B) Identify of differentially abundant proteins (DAPs) including their functions and fold change. C) Protein-Protein interaction (PPI) network using the list of the 16 DAPs in Ross 308 chicken breast muscle. The PPI network was built using the STRING database (https://string-db.org/). D-E) Bioinformatics enrichment analyses (Gene Ontology, KEGG, Reactome) using the 16 DAPs. D) Top enriched cluster terms. E) Biological processes. The enrichment analysis was performed using Metascape webservice tool (https://metascape.org/).

The overlap of 2-DE gels of Ross 308 chicken meat from organic (ORO) and antibiotic-free (ARO) farming systems and the 16 differentially abundant spots are shown in Fig. 4A. Gene names, Uniprot ID, protein names and functions are reported in Fig. 4B together with their fold change values calculated in 2-DE image analysis. Glycerol-3-phosphate dehydrogenase (GPD1), troponin I (TNNI2) and Tau-tubulin kinase 1 (TTBK1) were more abundant in organic samples while ACTA2, phosphoglycerate kinase (PGK2) and MYH2 were the main overabundant proteins in antibiotic-free samples. These proteins can be investigated as biomarkers to discriminate organic and antibiotic-free chicken meat production system from Ross 308 strain.

The PPI analysis highlighted one main network composed by nine proteins related to muscle structure pathways (Fig. 4C). The proteins with highest number of interactions were ACTA1 with 5 interactions and ACTN2 and MYH2 with 4 each. The GO enrichment analyses on the 16 DAPs through Gene Ontology, KEGG and Reactome databases are given in Fig. 4D and Fig. 4E. It resulted that 6 GO cluster terms were significantly enriched mainly dominated by “muscle contraction (GO:0006936)” and “mesenchyme migration (GO:0090131)”. Furthermore, 12 biological processes were enriched with “multicellular organismal process (GO:0032501)”, “developmental process (GO:0032502)” and “metabolic process (GO:0008152)” as the top enriched biological processes terms.

### 3.4 Ranger Classic chicken meat samples: DAPs between farming systems

**Fig. 5:**
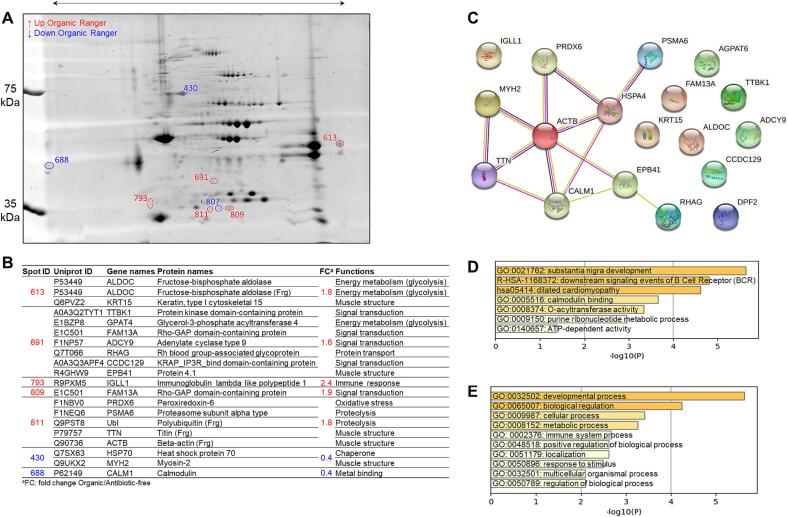
Comparison between antibiotic-free and organic chicken breast muscle proteomes within the Ranger Classic strain. A) Representative two-dimensional gel electrophoresis highlighting the differentially abundant protein spots between the two groups. Spots in blue color correspond to the down-regulated proteins, those in red are up-regulated proteins in organic chicken meat. B) Identify of differentially abundant proteins (DAPs) including their functions and fold change. C) Protein-Protein interaction (PPI) network using the list of the 18 DAPs in Ranger Classic chicken breast muscle. The PPI network was built using the STRING database (https://string-db.org/). D-E) Bioinformatics enrichment analyses (Gene Ontology, KEGG, Reactome) using the 16 DAPs. D) Top enriched cluster terms. E) Biological processes. The enrichment analysis was performed using Metascape webservice tool (https://metascape.org/).

The last comparison is related to the overlap of 2-DE gels of Ranger Classic chicken meat from organic (ORA) and antibiotic-free (ARA) farming systems, which identified 8 differentially abundant spots (Fig. 5A). The gene names, Uniprot ID, protein names and functions are reported in Fig. 5B together with their fold change values calculated within the 2-DE images. Among the proteins, IGLL1, FAM13A and fructose-bisphosphate aldolase (ALDOC) were more abundant in organic breast meat samples while heat shock protein 70 (HSP70), MYH2 and CALM1 were the most abundant DAPs in antibiotic-free samples. These proteins can be proposed as biomarkers to discriminate between organic and antibiotic-free chicken farming system from Ranger Classic chicken strain.

As for the previous comparison for Ross 308 chicken, the PPI analysis highlighted one main network composed on nine proteins related to muscle structure pathways (Fig. 5C). The proteins with highest number of interactions were ACTB with 6 interactions and CALM1 and HSPA4 with 4 each. The GO enrichment analyses on the 18 DAPs through Gene Ontology, KEGG and Reactome databases are given in Fig. 5D-E. It resulted that 7 GO cluster terms were significantly enriched mainly dominated by “substantia nigra development (GO:0021762)”, “downstream signaling events of B cell receptor (BCR) (R-HSA-1168372)” and “dilated cardiomyopathy (hsa:05414)”. Furthermore, 10 biological processes were enriched with “developmental process (GO:0032502)”, “biological regulation (GO:0065007)” and “cellular process (GO:0009987)” as the top enriched biological processes terms.

### 3.5 Summary of the results and proteins of high importance within strains and farming systems

**Fig. 6:**
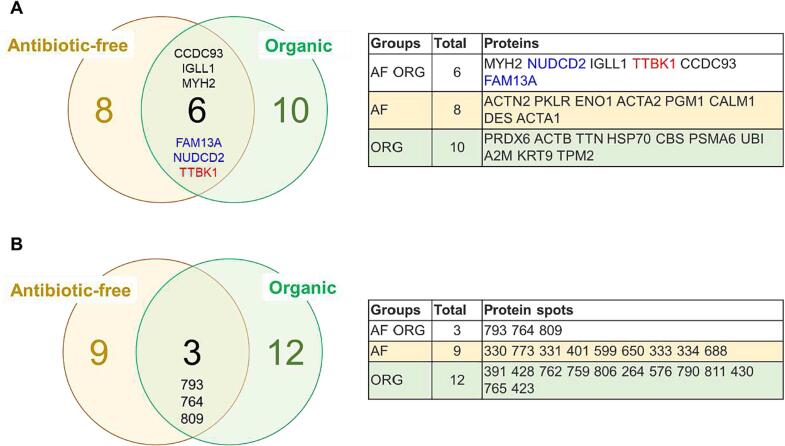
Venn diagrams related to antibiotic-free (AF) and organic (ORG) chicken breast meat samples. A) Venn diagram highlighting the number of common proteins. In blue down-regulated and in red up-regulated proteins in Ranger Classic chickens compared to Ross 308 strain. B) Venn diagram related to the identified protein spots.

**Fig. 7:**
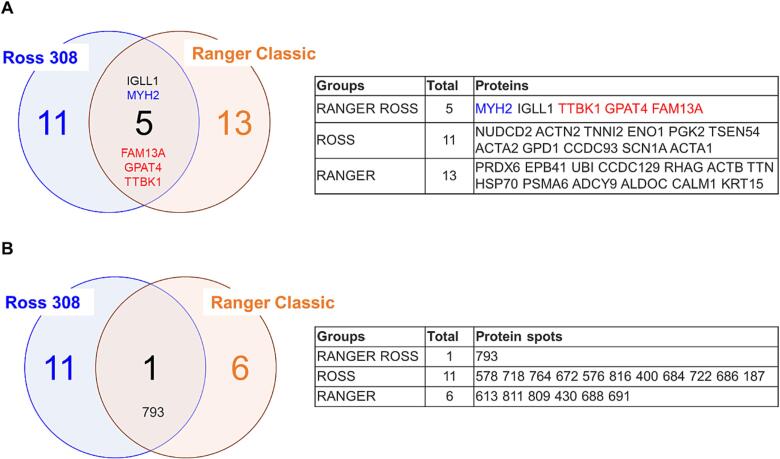
Venn diagrams comparing Ross 308 and Ranger Classic chicken breast meat samples. A) Venn diagram highlighting the number of common proteins. In blue down-regulated and in red up-regulated proteins in organic samples compared to antibiotic-free. B) Venn diagram related to the identified protein spots.

An overview on DAPs among chicken breasts from the antibiotic-free and organic farming systems is reported in Fig. 6. We identified six proteins to be common, from which three proteins were in the same direction (trend) within strain comparison. NUDCD2 and FAM13A were overabundant and TTBK1 was less abundant in Ranger Classic samples. The DAPs among Ross 308 and Ranger Classic chicken breasts are reported in Fig. 7. A total of five proteins were in common and four of them has the same trend in farming system comparison. Among these, MYH2 was down-regulated and FAM13A, GPAT4 (Glycerol-3-phosphate acyltransferase 4) and TTBK1 were overabundant in organic samples.

**Fig. 8:**
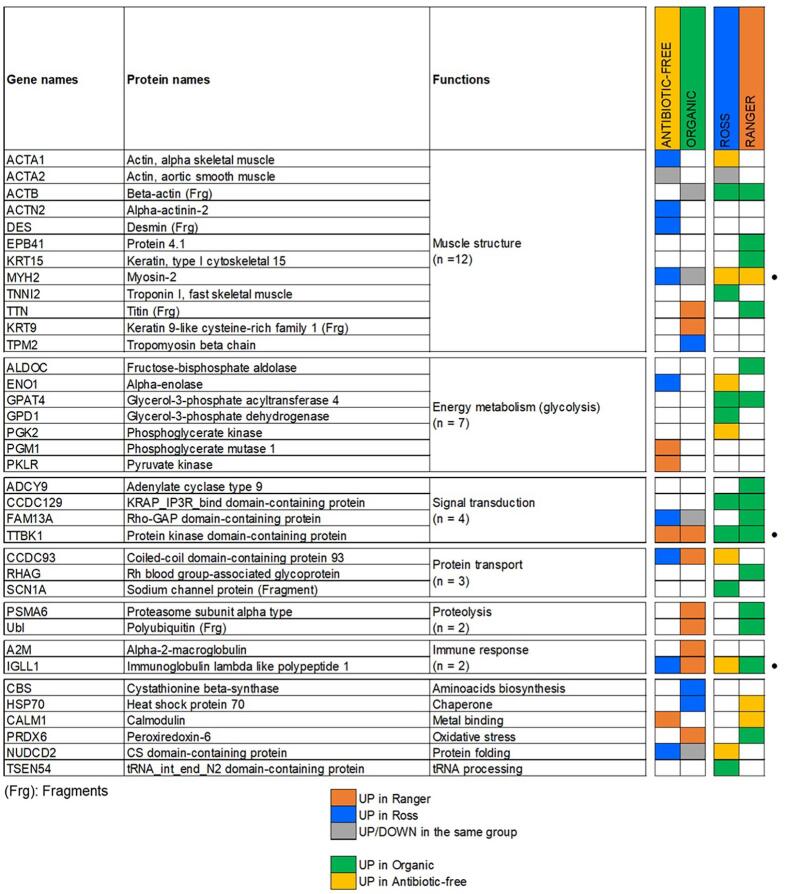
Summary of the differentially abundant proteins (DAPs) organized by molecular function identified in this study to differ among the factors. The proteins identified whatever the condition are shown in the right of the panel with a symbol. The symbol (Frg) indicates that fragments of that protein were quantified.

In Fig. 8, we summarized the entire changing proteins, the direction of their abundances among the four experimental groups and the molecular pathways to which they belong. MYH2, IGLL1 and TTBK1 were identified as DAPs whatever the condition (farming system and chicken strain). MYH2 was overabundant in antibiotic-free chicken meat, especially in Ross 308. TTBK1 was overabundant in organic and in Ranger Classic samples. IGLL1 expression seemed to be influenced by both farming system and chicken strain. Interestingly, this protein seemed to be overabundant in Ross 308 when comparing antibiotic-free farming system and in Ranger Classic when comparing organic farming systems.

## 4 Discussion

This study aimed to increase our knowledge on chicken meat (muscle) proteome, by considering for the first-time chicken strains and farming systems as factors of variations. The results of 2-DE and LC-MS/MS analyses evidenced the multiple changes that occur in the post-mortem muscle proteome within chicken strains and farming conditions. Specific proteomic signatures resulted from the differentially expressed proteins and they further provided clues on the biochemical processes that might influence meat quality traits. Moreover, the results would allow proposing a list of candidate protein biomarkers as indicators of meat authenticity.

Several research groups investigated the proteome of chicken breast meat using 2-DE followed by LC-MS/MS analysis to explore several aspects of poultry meat research including breast abnormalities and meat quality defects (Kuttappan et al., 2017, Cai et al., 2018, Zhang et al., 2020a, Zhang et al., 2020b, Zhang et al., 2021). However, there are no scientific evidence about the relation between chicken muscle proteome to the type of farming system and very few on the emerging chicken strains used by the industry (Dal Bosco et al., 2021). In the following sections, we discussed the important results from this preliminary proteomics study and compared them to the few available studies in the field in the literature.

### 4.1 Differences in the muscle proteomes of organic *versus* antibiotic-free chicken farming systems

The characterization of the post-mortem muscle proteome of chicken breast meat revealed notable differences between organic and antibiotic-free farming systems whatever the strains. Interestingly, antibiotic-free samples revealed a higher abundance of proteins related to muscle contraction. In particular, the comparison of Ross 308 strain breast showed a statistically relevant influence of muscle contraction pathway as it was the major enriched term in the GO enrichment analysis. Sex, slaughter age of birds and their live weight at slaughtering was associated with different meat content and composition (Diaz et al., 2010). The modification on the muscle characteristics can be further related to the growth of the animals, which is in line with the higher slaughter weight of the animals of Ross 308 strain compared to ranger irrespective of the farming system. Indeed, the difference in terms of lifespan between organic and antibiotic-free meat is an important gap, moreover, Ross 308 chicken strain is known to have faster grow rate than Ranger Classic (Suliman et al., 2023). In line to the findings of our study, earlier reports evidenced muscle proteome dynamics changes analyzed under conditions of rapid growth (Doherty et al., 2004).

In this study, antibiotic-free broilers were found to be able to grow faster and they are slaughtered earlier than those raised under organic farming system. This can be exemplified by the abundances of several proteins in antibiotic-free meat such as ACTA2, as one of the important components of the muscle contractile apparatus (Gagaoua et al., 2021). In line with our study, ACTA2 have been reported to be a key player in the growth of breast muscle of Ross 308 compared to native chickens (Albooshoke et al., 2018). CALM1, in the form of calmodulin-calcium complex, is another key component of the cGMP-PKG and calcium signaling pathways (Liu et al., 2020). It is a protein of interest due to the stimulating role of several enzymes, such as myosin light-chain kinases and phosphatases, all responsible of the energy-related muscle contraction (Crow and Kushmerick, 1982, Isotani et al., 2004, Stull et al., 2011). Furthermore, and in support of our findings on muscle structure dynamics, CALM1 is a vital component in the regulation of muscle fiber type transformation (Young and Leighton, 1998, Mallinson et al., 2009). Interestingly, recent data on target validation reported that CALM1 was a target of gga-miR-196-5p and the authors hypothesized that such miRNA could target sequences in CALM1 genes to regulate the chicken muscle fiber phenotype (Liu et al., 2020). In support of these results, MYH2, a myosin isoform involved in muscle contraction mechanisms (Tojkander et al., 2011) was further identified as a key separating protein. In fact, MYH2 was retained in both the ARO-ORO and ARA-ORA comparisons, being thus a good indicator for farming system authentication, being overabundant in antibiotic-free meat irrespective of the chicken strain (especially in Ross 308). MYH2 is involved in ventral stress fibers contraction responsible of cell shape changes during cell migration and in cell borders structure against inward membrane pressure (Tojkander et al., 2011). Together with actin (ACTA1), phosphoglycerate kinase (PGK), creatine kinase M type (CKM), beta-enolase (ENO3), carbonic anhydrase 2 (CA2), proteasome subunit alpha (PSMA4), pyruvate kinase (PKM), and malate dehydrogenase (MDH2), MYH2 was reported to be more expressed in chicken breasts affected by PSE abnormality (Desai et al., 2016). Previous research on pig meat showed that MYH2 expression contributed to increase in muscle mass, whereas the presence of oxidative fibers was favorably correlated with meat color features, greater water-holding capacity and improved meat tenderness (Wimmers et al., 2008). These important functions are worthy to explore further with the aim of validating these proteins as putative biomarkers. Moreover, a better understanding on how chicken muscles grow and adapt as a function of farming system and targeted strain could help the producers provide the optimal growth conditions to control the final quality of the products in line with the sustainability objectives of producing better with less resources.

Proteins related to energy metabolism pathways (GPAT4, GPD1 and ALDOC) and signal transduction (FAM13A and TTBK1) resulted up-regulated in organic chicken breasts while the proteins of muscle contraction pathways (ACTA2, MYH2 and CALM1) were up-regulated in antibiotic-free ones. From the few studies available in terms of the relationships between *post-mortem* muscle proteomes and meat quality of broilers, the expression of these proteins can be related to a better intrinsic meat quality and to absence of myopathic lesions. In fact, an earlier work by Kuttappan and co-workers compared the proteomic profiles of normal breasts with breasts that report severe myopathic lesions (Kuttappan et al., 2017). The results showed that biochemical pathways related to an increase in protein synthesis and cellular stress were up-regulated in breasts with severe lesions while others related to energy metabolism such as glycolysis and gluconeogenesis were up-regulated in normal breasts. Another study underlined decreased glycolytic activity and increased activation of specific pathways in response to oxidative stress in woody breast meat (Cai et al., 2018).

In the present study, FAM13A and TTBK1 were identified for the first time in chicken muscles to play a role in both Ross 308 and Ranger Classic, irrespective of the farming system. These two proteins were grouped in the signal transduction pathway. FAM13A is a Rho-GAP domain-containing protein involved in the phosphorylation of GTPases, in GTP hydrolysis and also in body fat distribution and adipocyte function (Fathzadeh et al., 2020). FAM13A has been reported using transcriptomics and antibody-based proteomics to be highly expressed in human adipose tissue (Fagerberg et al., 2014), however, nothing is yet known for poultry and this deserves a comprehensive study. Further, the biological function of FAM13A in the context of our study related to the discrimination of farming systems and chicken strains is not fully understood and future studies are necessary to decipher the mechanistic roles this protein would play including for chicken meat quality. The protein TTBK1, which is a Tau-tubulin serine/threonine kinase able to phosphorylate the TAU protein on serine, threonine and tyrosine residues by inducing aggregation (Nozal & Martinez, 2019), was one of the top three proteins that were changing whatever the condition. TTBK1 was in this study overabundant in organic and in Ranger Classic samples. This protein is the first isoform of tau-tubulin kinase (TTBK) and an its enhanced expression or activation was proposed to lead to increased tau phosphorylation (Ikezu & Ikezu, 2014). Further studies are necessary to characterize TTBK1 in the context of poultry research.

Among the proteins retained for Ross 308 and Ranger Classic groups, GPAT4 protein emerged as up-regulated specifically in organic samples. GPAT4 is a member of the Glycerol-3-phosphate acyltransferase family involved in the phospholipid metabolism and acts on the first step in the glycerophosphate pathway (Wendel et al., 2009). *GPAT* gene family is catalyzing the conversion of glycerol-3-phosphate and acyl-CoA to form 1-acylglycerol-3-phosphate. GPAT4 is the fourth isoform of GPAT family members that has been discovered (Nagle et al., 2008) and was found to be located exclusively in the endoplasmic reticulum with an expressed protein of 48 kDa (Beigneux et al., 2006). GPAT4 has been shown to use a range of acyl-CoA substrates from 12 to 20 carbons, however the highest GPAT activity seemed to occur with acyl-CoA species of 16- and 18-carbons, whatever the level of saturation (Nagle et al., 2008). We suppose that it is through the regulation of triacylglycerol (TAG) synthesis and metabolism with important roles in whole body energy homeostasis that this protein is involved making it based on our results as a specific molecular signature of organic farming system. In poultry, nothing is known about the GPAT members in relation to meat quality, however, recent studies demonstrated their important roles based on liver chicken (Yang et al., 2019) and in studies on other species evidenced the impact of *GPAT1* gene polymorphisms on fat deposition traits and fatty acids composition of cattle (Jeong et al., 2012, Yu et al., 2017) and pork (Mitka et al., 2020).

Finally, the last protein of interest these being IGLL1, which was found to be influenced by both farming system and chicken strain (Fig. 8). Interestingly, this protein seemed to be overabundant in Ross 308 when comparing antibiotic-free farming system and in Ranger Classic when comparing organic farming systems. IGLL1 is an immunoglobulin critical for B-cell development, so, linked to the immune response and inflammation (Hollis et al., 1989, Ohnishi et al., 2000). The expression of this protein would be explained by an effective immune system in the broilers and B-cell development through the recombination of immunoglobulin light chain loci (Benatar et al., 1992). Interestingly, IGLL1 seemed to be overabundant in Ross 308 when comparing antibiotic-free farming system and in Ranger Classic when comparing organic farming systems. Given the substantial differences in the two farming systems involving lifespan and gender, it can be hypothesized that each of the two strains we considered is optimal for a specific farming. In fact, fast-growing chicken breasts (Ross 308) express this immunoglobulin when raised in an antibiotic-free manner while slower-growing chickens (Ranger Classic) express it if raised organically. This major finding needs targeted studies to decipher the underlying mechanisms.

### 4.2 Differences in the muscle proteomes of Ross 308 *versus* Ranger Classic chicken strains

In the antibiotic-free chicken meat comparison, a high number of the DAPs belonging to the energy metabolism pathways were found to be overabundant in Ranger Classic chicken while muscle structure proteins were overabundant in Ross 308 both from antibiotic-free farming system. It is interesting that this difference does not emerge from the comparison of organic farming where Ranger Classic strain expressed more immune system related proteins compared Ross 308. Such findings can be ascribed to the differences that might originate from the sex and age at slaughter of the broilers. Accordingly, a recent study investigated the impact of chicken age and sex on the amino acid and fatty acid profiles of muscles from fast-growing Ross 308 broiler (Suliman et al., 2023). The authors reported that meat nutritional values can be affected by slaughter age more than by sex. In fact, our chicken strain comparison involved animals of both sex (females for organic farming and males for antibiotic-free farming) resulting in high impact of chicken strain in muscle proteome.

Among the muscle structure proteins, MYH2 previously discussed was further identified among the DAPs in the comparison of the two strains. This is one of the three proteins highlighted in Fig. 8 as identified in all the conditions. In particular, it emerged as up-regulated in ARO samples when compared to ARA. The obtained results can be related to the faster-growing of Ross 308 chickens that probably reach an optimal muscle structure in the lower lifespan characteristic of antibiotic-free farming system. In organic samples, MYH2 was highly expressed in both chicken strains, but with no statistical difference. It leads to remarking that giving a longer lifespan, also Ranger Classic chickens can achieve the desirable growth and muscle structure. A published research by De Liu et al. (2012) that compared Korean native chicken meat and conventional broilers, identified MYH as putative biomarker in strain authentication. In line with our results, they set a different lifespan within the strains (35 days for conventional broilers and 77 days for Korean native chickens), so that both strains, being fast and slow-growing respectively, could reach a mature muscle structure. Moreover, they stated that breed accounts for most factors affecting the muscle fiber composition in line to our findings. Further targeted studies are worthy to consider to better explore the underlying mechanisms. The use of other high-throughput proteomics studies such as shotgun proteomics (Álvarez et al., 2023, Lamri et al., 2023a(della Malva, Lamri, Albenzio, & Gagaoua, 2023c)) to accurately evaluate the total or myofibrillar, sarcoplasmic and mitochondrial sub-proteomes of the chicken muscles.

Energy metabolism related proteins were mainly identified to change for Ranger Classic strain chickens within the antibiotic-free comparison, and identified in four of six protein spots that were differentially abundant. The main recurrent protein is PGM1 (phosphoglucomutase 1), an enzyme involved in glycolysis and glycogenesis (the process of glycogen synthesis) as it catalyzes the interconversion of glucose-1-phosphate (G-1-P) and glucose-6-phosphate (G-6-P)) (Greenberg et al., 2006). These processes are intense metabolic activities for the cells so they are expected in muscles with a high amount of fast twitch myofilaments (Picard and Gagaoua, 2020b, Gagaoua et al., 2021). More specifically and during the transformation of the muscle into meat, an imbalance of the two substrates of PGM1 (G-1-P and G-6-P) occurs. Excess G-1-P is produced due to the breakdown of glycogen while available G-6-P is used for glycolysis to produce ATP (Greenberg et al., 2006). Thus, PGM1 is a vital glycolytic enzyme in providing G-6-P for the production of ATP from glycogen in both living and early post-mortem muscles. In consistent with the type of muscle we investigated in this study (white chicken meat), the activity of PGM1 was reported to be lower in oxidative fibers of skeletal muscle (red meat) as they mainly use the anaerobic metabolism to generate the required fuel for energy production. The presence of these muscle fibers could be related to animals with an active life in terms of movements as they are suited to activities that involve short bursts of power, thereby generating a similar muscle contraction force as slow-twitch fibers but at a more rapid rate (Bouley et al., 2005). Besides the role of PGM1 in glucose metabolism regulation, it was also ascribed to play a role in muscle development, while its deficiency results in congenital dysglycosylation and probably lower glycogen storage that can be manifested by short stature (Bae et al., 2014). Refers to meat quality, PGM1 is a well-known biomarker of poultry meat quality traits (Zheng et al., 2014, Cai et al., 2018) and for other meat species such as pork and beef (Gagaoua et al., 2020, Lopez-Pedrouso et al., 2020).

Referring to the study by De Liu et al. (2012), PGM1 expression was correlated to a high glycolytic metabolism and high glycogen level in Korean native chickens with regard to commercial broilers. The authors evidenced that the increase of glycolytic metabolism was further associated with the up-regulation of the small heat shock protein HSP27 (De Liu et al., 2012). In contrast with this, the expression of heat shock proteins in our results emerged as statistically higher in Ross 308 reared under the organic farming comparison. However, it is the large heat shock protein HSP70 which was identified in three of the seven differentially abundant spots. HSP70 is an inducible stress protein involved in the protection of the cell from external stress stimulus as it plays a pivotal role in the protein quality control system, ensuring the correct folding of proteins, the re-folding of misfolded proteins and controlling the targeting of proteins for subsequent degradation (Mayer, 2013, Gagaoua et al., 2015, Gagaoua et al., 2017). Referring to the overabundance of HSP70 could allow us hypothesize that the longer lifespan of fast-grow rate chicken can be a cell stress-inducing factor. This idea is also supported by an over-expression of the folding protein NUDCD2 in the same samples for which its function was ascribed to be related to HSP90 chaperone stabilization (Taipale et al., 2014).

The proteins related to proteolysis (UBI and PSMA6) were significantly overabundant in organic Ranger Classic compared to organic Ross 308. Polyubiquitin chains link target proteins to start protein degradation and they were reported to be regulated by feeding status (Seiliez et al., 2008). PSMA6 is an ATP-dependent proteasome involved in the degradation of ubiquitinated proteins (Naujokat & Hoffmann, 2002). The identification of these major proteins with key roles in apoptosis, autophagy and proteolysis would lead to differences in the final quality of the two strains that are worthy to explore in the future. An overexpression of proteins involved in apoptosis, protein synthesis, and oxidative stress were for example related to the occurrence of woody breast meat (Zhang et al., 2020a). Further studies are needed to explore the relationships between pathways related to apoptosis and autophagy and their consequences in the development and/or determination of the intrinsic meat quality traits from the chickens strains were investigated within the two farming systems we considered.

## 5 Conclusions

The differences in strains and farming system seemed to highly influence the chicken breast post-mortem muscle proteome with possible impact on meat quality. Comparing the results with scientific literature, it was found that specific differences in the early post-mortem chicken proteome due to the disparities in broiler genetic can lead more likely to the emergence of meat with different qualitative traits. Moreover, this study focused on organic and antibiotic-free farming systems, underlining for the first time the differences in protein expression in chicken breast. The lifespan resulted to be a crucial difference between the two farming methods allowing us to hypothesize that the antibiotic-free Ranger Classic chickens maybe lacks enough time to develop a mature muscle structure and a ready immune system, which instead was reported when they are raised organically so with longer lifespan. At the same time, the Ross 308 chickens developed these features more quickly according to their fast-growing strain. This aspect should be more investigated by further research as a paucity of scientific literature was found about the impact of organic production system on chicken breast muscle proteome. This study evidenced the first molecular signatures at interplay between the differences that exist between farming systems or strains. Further studies using high-throughput proteomics studies such as shotgun proteomics would allow decipher in-depth the underlying mechanisms and propose biomarkers for both the authenticity of the origin and of chicken meat quality traits.

## CRediT authorship contribution statement

**Laura Alessandroni:** Writing – original draft, Investigation, Methodology, Formal analysis, Visualization, Data curation, Conceptualization. **Gianni Sagratini:** Writing – review & editing, Validation, Supervision, Project administration, Funding acquisition, Conceptualization. **Mohammed Gagaoua:** Writing – review & editing, Writing – original draft, Visualization, Validation, Supervision, Software, Resources, Methodology, Investigation, Formal analysis, Data curation, Conceptualization.

## Declaration of competing interest

The authors declare that they have no known competing financial interests or personal relationships that could have appeared to influence the work reported in this paper.

## Data Availability

Data will be made available on request.
